# Prognostic analysis of hidradenocarcinoma: a SEER-based observational study

**DOI:** 10.1080/07853890.2022.2032313

**Published:** 2022-02-02

**Authors:** Teng Gao, Sheng Pan, Meng Li, Runping Su

**Affiliations:** a Department of Dermatology, Changzhou Geriatric Hospital Affiliated to Soochow University, Changzhou No.7 People's Hospital, Changzhou, China; b Department of Neurology, Changzhou Geriatric Hospital Affiliated to Soochow University, Changzhou No.7 People’s Hospital, Changzhou, China

**Keywords:** Hidradenocarcinoma, prognosis, SEER, observational study

## Abstract

**Background:**

Hidradenocarcinoma is a rare malignancy of sweat gland differentiation. Published literature has reported that hidradenocarcinoma has a high recurrence and metastasis rate, and the prognosis is extremely poor. However, the sample sizes included in these studies are insufficient, and therefore, the findings are doubtful.

**Materials and methods:**

Clinicopathological characteristics and survival data of 289 hidradenocarcinoma patients were extracted from the SEER database (covering 18 registries, 2000–2018) released in July 2021. The distribution of clinicopathological characteristics was compared using the Pearson chi-square test. Overall survival (OS) and cancer-specific survival (CSS) were analysed using the log-rank test and univariate analysis.

**Results:**

The primary site of hidradenocarcinoma in 121 patients was located in the head and neck, accounting for 41.9%, and the others were located in the trunk and limbs. For hidradenocarcinoma, the mean OS and CSS were 164 months and 165.9 months, respectively; the 10-year OS rate and CSS rate were 60.2% and 90.5%, respectively. Survival analysis showed that the primary site, sex, age, race, histologic grade, stage, and surgery are not associated with hidradenocarcinoma patients’ OS or CSS. For head and neck hidradenocarcinoma or trunk and limbs hidradenocarcinoma, sex, age, race, histologic grade, AJCC stage, and primary site surgery are still not related to prognosis. Tumour size is correlated with patients’ OS rather than CSS.

**Conclusions:**

Hidradenocarcinoma is a malignant tumour with a good prognosis, which is different from previous views. Tumour size is inversely proportional to patients’ overall survival time affecting the OS and CSS of patients. Improving health awareness, initial histological examination and timely surgery are the keys to improving the prognosis.

## Background

In 1954, Keasby and Hadley first reported that hidradenocarcinoma was believed to be an uncommon malignant transformation of nodular hidradenoma [1]. As an uncommon malignant transformation, hidradenocarcinoma was found in 1:13,000 skin biopsies, accounting for only 6% of malignant eccrine tumours [2,3]. The study by Kazakov et al. [4] showed that a hidradenoma remnant was found in 5 of 14 primary hidradenocarcinomas, and the transition to a cancerous component was clearly evident. Hidradenocarcinoma is also known as malignant clear cell hidradenoma, clear cell hidradenocarcinoma, and malignant acrospiroma [5,6]. The main clinical manifestations of hidradenocarcinoma are isolated, hard, asymptomatic intradermal erythematous, or violaceous nodules on the head, neck, trunk, limbs, or oral cavity [7]. The accepted histological criteria for diagnosing hidradenocarcinoma include lack of demarcation, invasive growth pattern, deep extension, nuclear pleomorphism, necrosis, vascular invasion, perineural invasion, and the presence of an increased number of mitoses [8–10]. Hidradenocarcinoma usually grows slowly for many years, but it may increase rapidly in the short term [11].

At present, there are only a few clinical studies about hidradenocarcinoma [12–15]. In these studies with limited case numbers and follow-up, an aggressive clinical course characterised by local recurrence and systemic metastases is frequently observed [1,4,15–17]. The study conducted by Souvatzidis et al. reported a series of 7 hidradenocarcinoma patients and observed their clinicopathological characteristics and biological manifestations [15]. They concluded that hidradenocarcinoma has a high recurrence and metastasis rate, and the prognosis is extremely poor. Nazarian et al. [16] followed up 8 hidradenocarcinoma patients (range 2 months to 3 years, median 1 year and 5 months). Multiple regional lymph nodes and skin metastases were observed in 2 patients at 8 months and 3 years after resection, respectively. Kazakov et al. [4] followed 12 hidradenocarcinoma patients (range 4 months to 11 years, median 3.5 years). Two patients died of multiple visceral metastases at 4 months and 5 months after diagnosis, respectively. Of the remaining 10 surviving patients, a local recurrence was observed in 3 patients of whom one experienced 2 local recurrences. To better assess the prognosis of hidradenocarcinoma, we conducted a systematic analysis of the hidradenocarcinoma data in the Surveillance, Epidemiological and End Results (SEER) database.

## Materials and methods

### Data retrieval

This research was approved by the Ethical Committee of Soochow University and followed the guidelines outlined in the Declaration of Helsinki. After obtaining the research permission (serial number: 14414-Nov2020), we used SEER* Stat version 8.3.9.1 to retrieve the SEER database (covering 18 registries, 2000–2018) released in July 2021. Because patient data come from a public database, informed consent was not required.

The selection variables were histological type (ICD-0–3) for malignant nodular hidradenoma (8402/3) and site recode (ICD-0–3/WHO2008) for other nonepithelial skin diseases. Patients with any other malignant tumours were excluded. The following variables were extracted from the database: cause of death, follow-up, sex, age, race, tumour size, histologic grade (ICD-0–2), American Joint Committee on Cancer (AJCC) stage, and primary site surgery.

### Statistical analysis

Except for survival data, other data were converted into categorical variables and compared with the Pearson chi-square test. Overall survival (OS) and cancer-specific survival (CSS) were derived from the cause of death provided by the SEER. The precise cut-off was defined according to the sensitivity, specificity and Youden Index [18]. The relationship between survival and follow-up was depicted as a Kaplan–Meier curve, and curves were compared using the log-rank test. Univariate analysis was used to screen the prognostic risk factors for hidradenocarcinoma, expressed in hazard ratios (HRs) and 95% confidence intervals (CIs). Multivariate analysis will be performed only when there is more than one variable with statistical significance (*p* < .05) in univariate analysis. When the *P* value was less than .05, the difference was considered to be statistically significant. SPSS version 22.0 statistical package (IBM, NY) and GraphPad Prism 8 (San Diego, California) was used for statistical analysis.

## Results

### Clinicopathological characteristics

Information on 289 hidradenocarcinoma patients was extracted from the SEER database, of which 170 were males, accounting for 58.8%. The mean age was 62.5 years (range 15–89 years). Table 1 summarises the distribution of the main characteristics. The incidence increased with age, with 60.6% of patients aged 60 years or older. White people account for the majority, while people of colour account for only 20.1%. The tumour size, histologic grade, and AJCC stage of many included patients are unknown. In patients with definite tumour size, the mean tumour size was 27.3 mm (range from 4 mm to 125 mm), the precise cut-off of tumour size for OS was 2.8 cm, and the precise cut-off of tumour size for CSS was 3.95 cm. In patients with known lymph node stage, the lymph node metastasis rate was 4.3% (10 of 235). In patients with known metastasis stage, the distant metastasis rate was 2.4% (6 of 248). Of the 6 patients with distant metastases, one had multiple bones and lung metastases, two had multiple brain and lung metastases, two had isolated lung metastases, and one had other (distant metastases, but none to bone, brain, liver, lung, and distant lymph node). The proportion of well-differentiated and moderately differentiated patients was nearly three times that of poorly differentiated and undifferentiated patients (21.1% versus 7.6%). The proportion of stage I–II patients (42.9%) was significantly higher than that of stage III–IV patients (6.6%). Only six patients (2.1%) had distant metastases at the time of diagnosis. A total of 90.3% of patients underwent primary site surgery after diagnosis.

**Table 1.** The distribution of the main clinicopathological characteristics of hidradenocarcinoma patients.

Variables	Number of patients	Primary site			
		Head and neck	Trunk and limbs	*χ* ^2^	*P* value
Sex				2.733	.098
Male	170 (58.8%)	78 (64.5%)	92 (54.8%)		
Female	119 (41.2%)	43 (35.5%)	76 (45.2%)		
Age				2.083	.353
0–29 years	12 (4.2%)	5 (4.1%)	7 (4.2%)		
30–59 years	102 (35.3%)	37 (30.6%)	102 (35.3%)		
≥60 years	175 (60.6%)	79 (65.3%)	175 (60.6%)		
Race				0.146	.702
White	231 (79.9%)	98 (81.0%)	133 (79.2%)		
Others	58 (20.1%)	23 (19.0%)	35 (20.8%)		
Tumour size				5.857	.439
1–10 mm	47 (16.3%)	25 (20.7%)	22 (13.1%)		
11–20 mm	36 (12.5%)	17 (14.0%)	19 (11.3%)		
21–30 mm	24 (8.3%)	9 (7.4%)	15 (8.9%)		
31–40 mm	23 (8.0%)	7 (5.8%)	16 (9.5%)		
41–50 mm	15 (5.2%)	5 (4.1%)	10 (6.0%)		
>50 mm	17 (5.9%)	5 (4.1%)	12 (7.1%)		
Unknown	127 (43.9%)	53 (43.8%)	74 (44.0%)		
Grade				0.361	.835
I–II	61 (21.1%)	27 (22.3%)	34 (20.2%)		
III–IV	22 (7.6%)	10 (8.3%)	12 (7.1%)		
Unknown	206 (71.3%)	84 (69.4%)	122 (72.6%)		
AJCC stage				0.655	.721
I–II	124 (42.9%)	54 (44.6%)	70 (41.7%)		
III–IV	19 (6.6%)	9(7.4%)	10(6.4%)		
Unknown	146 (50.5%)	58 (47.9%)	88 (52.4%)		
T stage				4.879	.087
T1–2	131 (45.3%)	52 (43.0%)	79 (47.0%)		
T3–4	18 (6.2%)	12 (9.9%)	6 (3.6%)		
Unknown	140 (48.4%)	57 (47.1%)	83 (49.4%)		
N stage				0.918	.632
N0	225 (77.9%)	97 (80.2%)	128 (76.2%)		
N1–2	10 (3.5%)	3 (2.5%)	7 (4.2%)		
Unknown	54 (18.7%)	21 (17.4%)	33 (19.6%)		
M stage				0.190	.909
M0	242 (83.7%)	102 (84.3%)	140 (83.3%)		
M1	6 (2.1%)	2 (1.7%)	4 (2.4%)		
Unknown	41 (14.2%)	17 (14.0%)	24 (14.3%)		
Primary site surgery				0.842	.359
Performed	261 (90.3%)	107 (88.4%)	154 (91.7%)		
Unperformed	28 (9.7%)	14 (11.6%)	14 (8.3%)		

AJCC: American Joint Committee on Cancer; T: Tumour; N: Node; M: Metastasis.

According to the primary site, we compared the patients into two groups: the head and neck and the trunk and limbs. The primary site of hidradenocarcinoma in 121 patients was located in the head and neck, accounting for 41.9%, and the others were located in the trunk and limbs. Table 1 shows the differences in the distribution of characteristics of patients with different primary sites. The results indicated that there was no significant difference in the distribution of sex, age, race, tumour size, histologic grade, AJCC stage, or primary site surgery between the two groups (*P* > .05).

### Survival analysis

**Figure 1.**
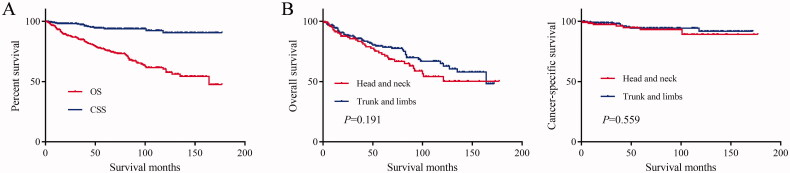
(A) Overall survival (OS) and cancer-specific survival (CSS) of hidradenocarcinoma patients. (B) Overall survival (OS) and cancer-specific survival (CSS) of hidradenocarcinoma patients stratified by primary site.

For all hidradenocarcinoma patients, the median follow-up time was 58 months. At the end of the follow-up, 84 patients died, of which 15 patients died of hidradenocarcinoma. The 1-year, 3-year, 5-year, and 10-year OS rates of these patients were 92.8%, 84.5%, 75.9%, and 60.2%, respectively (Table 2; Figure 1A). The mean and median OS was 122.8 months and 164 months, respectively (Table 2). The 1-year, 3-year, 5-year, and 10-year CSS rates of these patients were 98.2%, 97.2%, 93.8%, and 90.5%, respectively (Table 2; Figure 1A). The mean CSS was 165.9 months (Table 2).

**Table 2.** Survival rates of hidradenocarcinoma patients stratified by primary site.

Variables	All patients	Primary site		
		Head and neck	Trunk and limbs	*P* value
Overall survival				.191
1-year OS rate (95%CI)	92.8% (89.7%–95.9%)	91.3% (86.2%–96.4%)	93.8% (90.1%–97.5%)	
3-year OS rate (95%CI)	84.5% (79%–90%)	83.4% (76.3%–90.5%)	85.2% (79.5%–90.9%)	
5-year OS rate (95%CI)	75.9% (70.4%–81.4%)	72.6% (63.6%–81.6%)	78.1% (71.2%–85%)	
10-year OS rate (95%CI)	60.2% (52.6%–67.8%)	54.1% (41.9%–66.3%)	64.5% (54.9%–74.1%)	
Mean OS (months)	122.8	117	125	
Median OS (months)	164	–	164	
Cancer-specific survival	–	–	–	.559
1-year CSS rate (95%CI)	98.2% (96.6%–99.8%)	97.3% (94.4%–100%)	98.7% (96.9%–100%)	
3-year CSS rate (95%CI)	97.2% (95.0%–99.4%)	96.1% (92.2%–100%)	97.9% (95.5%–100%)	
5-year CSS rate (95%CI)	93.8% (90.5%–97.1%)	93.1% (87.6%–98.6%)	94.4% (90.3%–98.5%)	
10-year CSS rate (95%CI)	90.5% (85%–96%)	89.2% (80%–98.4%)	91.7% (85.2%–98.2%)	
Mean CSS (months)	165.9	163.9	162.7	
Median CSS (months)	–	–	–	

OS: overall survival; CSS: cancer-specific survival; CI: confidence interval.

For head and neck hidradenocarcinoma, the median follow-up time was 52 months. At the end of the follow-up, 38 patients died, of which seven patients died of hidradenocarcinoma. The 1-year, 3-year, 5-year, and 10-year OS rates of these patients were 91.3%, 83.4%, 72.6%, and 54.1%, respectively (Table 2). The mean OS was 117 months (Table 2). The 1-year, 3-year, 5-year, and 10-year CSS rates of these patients were 97.3%, 96.1%, 93.1%, and 89.2%, respectively (Table 2). The mean CSS was 163.9 months (Table 2).

For trunk and limbs hidradenocarcinoma, the median follow-up time was 62.5 months. At the end of the follow-up, 46 patients died, of which 8 patients died of hidradenocarcinoma. The 1-year, 3-year, 5-year, and 10-year OS rates of these patients were 93.8%, 85.2%, 78.1%, and 64.5%, respectively (Table 2). The mean and median OS were 125 months and 164 months, respectively (Table 2). The 1-year, 3-year, 5-year, and 10-year CSS rates of these patients were 98.7%, 97.9%, 94.4%, and 91.7%, respectively (Table 2). The mean CSS was 162.7 months (Table 2).

**Figure 2.**
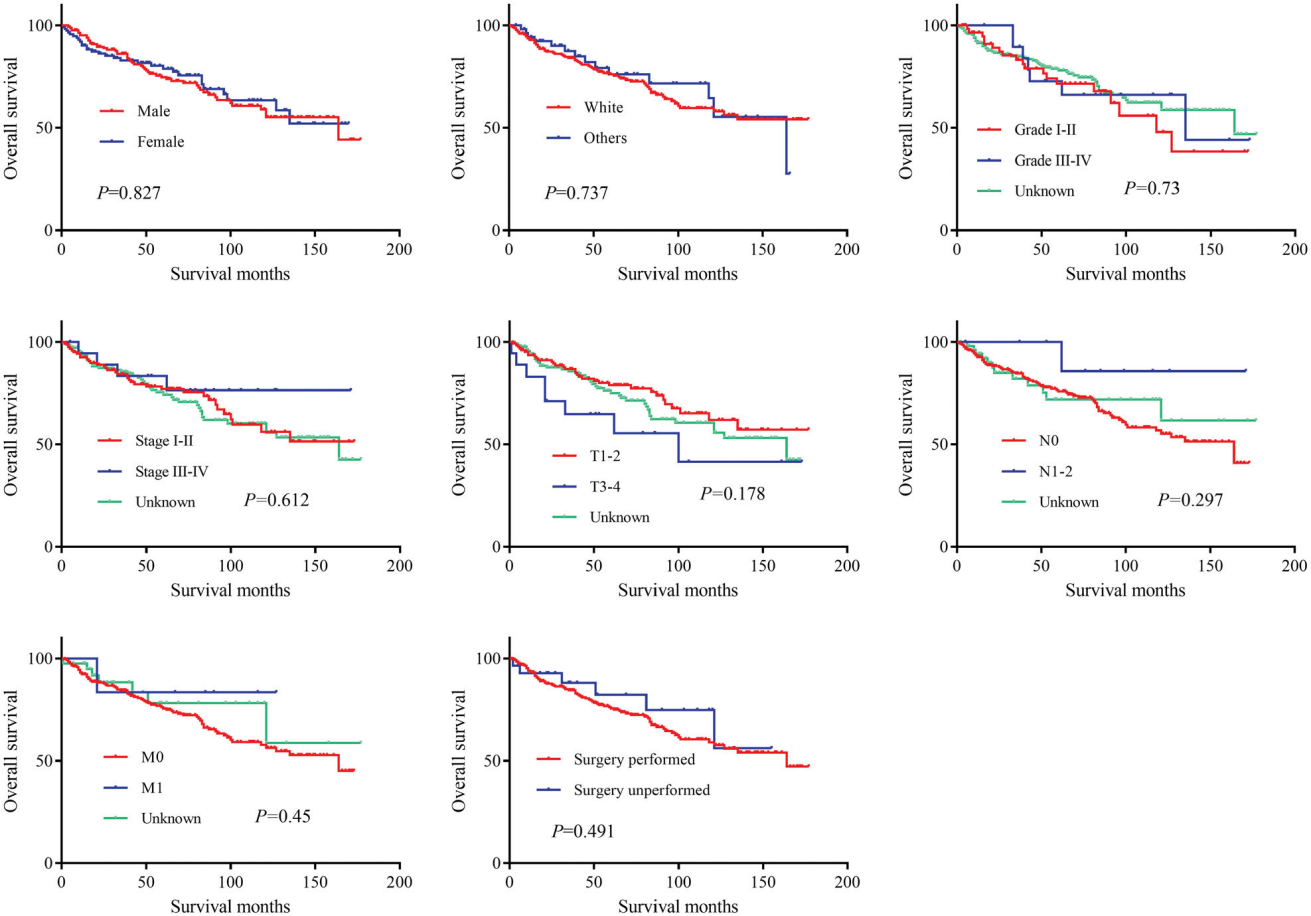
Overall survival (OS) of hidradenocarcinoma patients stratified by sex, race, histologic grade, American Joint Committee on Cancer (AJCC) stage, and primary site surgery.

**Figure 3.**
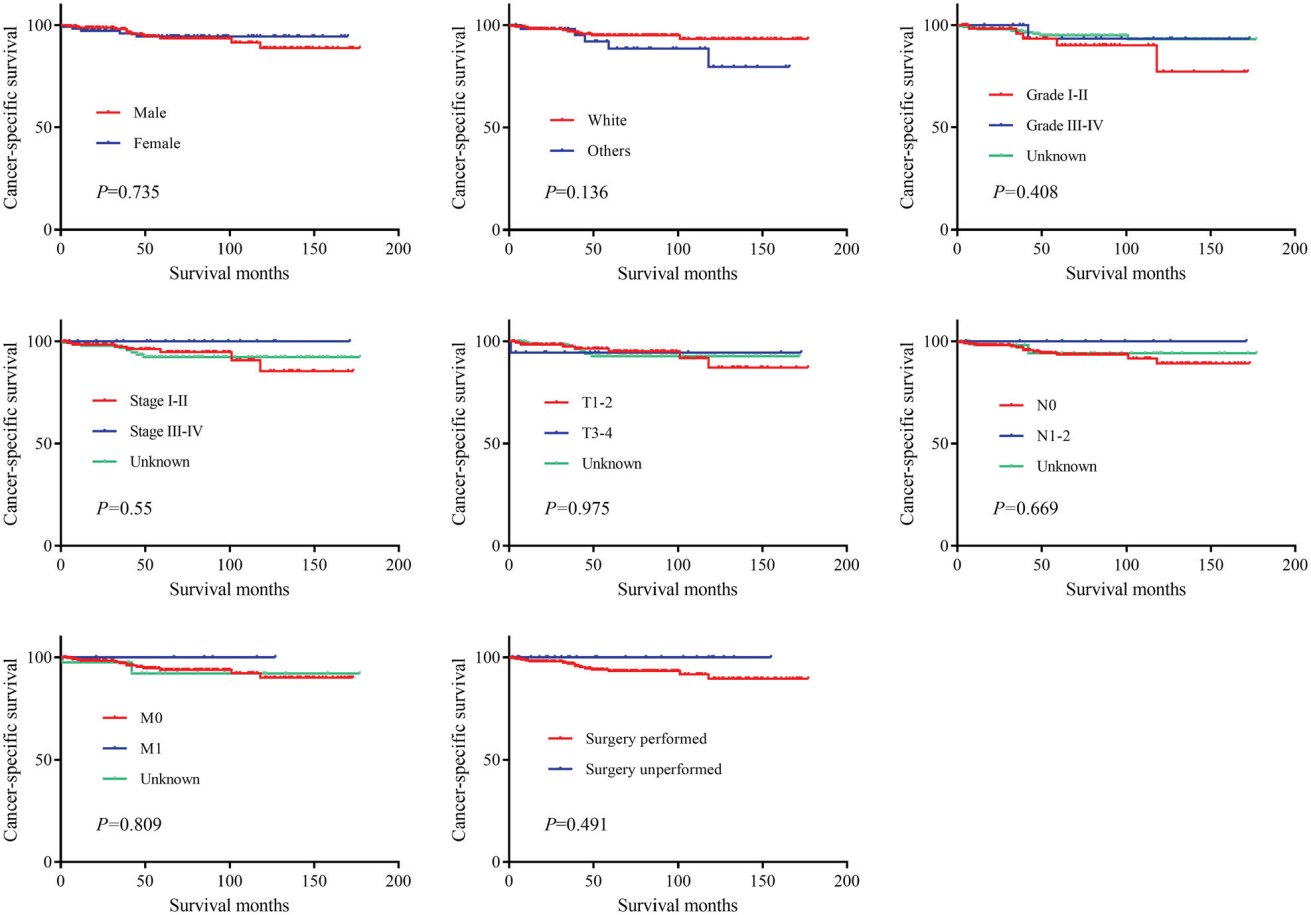
Cancer-specific survival (CSS) of hidradenocarcinoma patients stratified by sex, race, histologic grade, American Joint Committee on Cancer (AJCC) stage, and primary site surgery.

**Figure 4.**
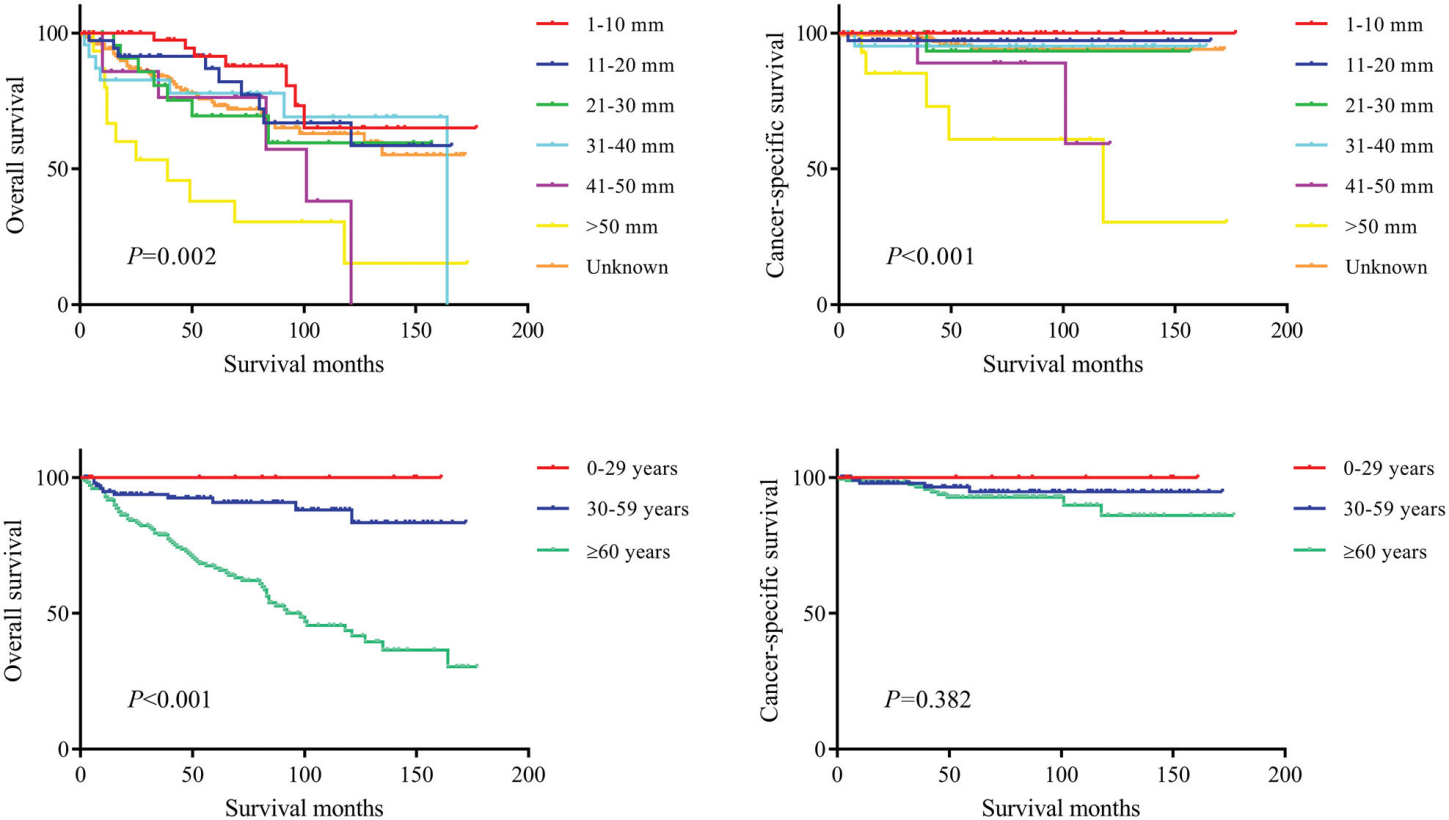
Overall survival (OS) and cancer-specific survival (CSS) of hidradenocarcinoma patients stratified by tumour size and age.

The log-rank test results showed that the primary site of hidradenocarcinoma was not a factor that affected patient OS (*p* = .191) or CSS (*p* = .559) (Table 2; Figure 1B). In addition, log-rank tests based on sex, race, histologic grade, AJCC stage, and primary site surgery also showed similar results (*P* > .05; Figure 2 and 3). However, tumour size is a factor that is related to the patient's prognosis. Hidradenocarcinoma patients with larger tumour sizes had worse OS (*p =* .002; Figure 4) and CSS (*P* < .001; Figure 4). Age appeared to be a factor related to patient OS (*P* < .001; Figure 4), but it was not related to patient CSS (*p* = .382; Figure 4).

The results of the univariate analysis showed that the primary site, sex, age, race, histologic grade, AJCC stage, and primary site surgery were not associated with patient OS or CSS (*P* > .05; Table 3). Tumour size was a factor related to the patient OS rather than CSS (Table 3), which was inconsistent with the log-rank test results. The patients were further grouped according to the primary site for univariate analysis. The results show that for head and neck hidradenocarcinoma or trunk and limbs hidradenocarcinoma, sex, age, race, histologic grade, AJCC stage, and primary site surgery were still not related to prognosis (*P* > .05; Table 4). Tumour size was still a factor related to the patient OS rather than CSS (Table 4).

**Table 3.** Univariable survival analysis of hidradenocarcinoma patients.

Variables	Overall survival		Cancer-specific survival	
	HR (95%CI)	*P* value	HR (95%CI)	*P* value
Primary site				
Head and neck	Reference		Reference	
Trunk and limbs	0.768 (0.499–1.182)	0.231	0.739 (0.267–2.044)	.561
Gender				
Male	Reference		Reference	
Female	0.981 (0.629–1.529)	0.933	0.831 (0.283–2.436)	.736
Age				
0–29 years	Reference		Reference	
30–59 years	–	0.909	–	.936
≥60 years	–	0.893	–	.931
Race				
White	Reference		Reference	
Others	0.892 (0.502–1.584)	0.697	0.218 (0.758–6.495)	.146
Tumour size				
1–10 mm	Reference		Reference	
11–20 mm	1.62 (0.603–4.354)	0.339	–	.934
21–30 mm	2.617 (0.949–7.221)	0.063	–	.93
31–40 mm	1.876 (0.657–5.36)	0.24	–	.932
41–50 mm	3.282 (1.102–9.774)	0.033	–	.922
>50 mm	6.297 (2.434–16.289)	<0.001	–	.916
Unknown	2.027 (0.902–4.557)	0.087	–	.931
Grade				
I–II	Reference		Reference	
III–IV	0.811 (0.342–1.92)	0.633	0.472 (0.055–4.054)	.494
Unknown	0.771 (0.463–1.284)	0.771	0.489 (0.163–1.461)	.2
AJCC stage				
I–II	Reference		Reference	
III–IV	0.629 (0.224–1.768)	0.379	–	.984
Unknown	1.044 (0.671–1.623)	0.85	–	.976
T stage				
T1–2	Reference		Reference	
T3–4	1.975 (0.913–4.273)	0.084	1.244 (0.153–10.114)	.839
Unknown	1.192 (0.758–1.875)	0.447	0.976 (0.342–2.788)	.964
N stage				
N0	Reference		Reference	
N1–2	0.255 (0.035–1.836)	0.175	–	.981
Unknown	0.805 (0.436–1.485)	0.487	0.748 (0.168–3.325)	.703
M stage				
M0	Reference		Reference	
M1	0.417 (0.058–3.001)	0.385	–	.985
Unknown	0.683 (0.314–1.482)	0.335	1.133 (0.255–5.039)	.87
Surgery				
Performed	Reference		Reference	
Unperformed	0.738 (0.321–1.694)	0.473	–	.413

HR: hazard ratio; CI: confidence interval; AJCC: American Joint Committee on Cancer; T: Tumour; N: Node; M: Metastasis.

**Table 4.** Univariable survival analysis of hidradenocarcinoma patients stratified by primary site.

Variables	Head and neck				Trunk and limbs			
	Overall survival		Cancer-specific survival		Overall survival		Cancer-specific survival	
	HR (95% CI)	*P* value	HR (95% CI)	*P* value	HR (95% CI)	*P* value	HR (95% CI)	*P* value
Gender								
Male	Reference		Reference		Reference		Reference	
Female	0.837 (0.394–1.777)	0.643	3.501 (0.768–15.963)	0.106	1.164 (0.652–2.077)	0.608	0.181 (0.022–1.473)	.11
Age								
0–29 years	Reference		Reference		Reference		Reference	
30–59 years	–	0.921	–	0.961	–	0.931	–	.967
≥60 years	–	0.91	–	0.96	–	0.914	–	.964
Race								
White	Reference		Reference		Reference		Reference	
Others	1.124 (0.494–2.555)	0.78	1.962 (0.379–10.157)	0.422	0.736 (0.329–1.647)	0.455	2.527 (0.603–10.596)	.205
Tumour size								
1–10 mm	Reference		Reference		Reference		Reference	
11–20 mm	1.855 (0.566–6.08)	0.308	–	1	1.47 (0.244–8.837)	0.674	–	.937
21–30 mm	0.563 (0.066–4.824)	0.6	–	1	6.532 (1.355–31.474)	0.019	–	.934
31–40 mm	3.989 (1.068–14.902)	0.04	–	0.956	1.607 (0.267–9.666)	0.605	–	1
41–50 mm	4.78 (1.276–17.912)	0.02	–	0.953	2.744 (0.386–19.505)	0.313	–	1
>50 mm	5.768 (1.365–24.368)	0.017	–	0.953	10.404 (2.202–48.147)	0.003	–	.923
Unknown	1.756 (0.638–4.833)	0.276	–	0.96	2.927 (0.684–12.529)	0.148	–	.941
Grade								
I–II	Reference		Reference		Reference		Reference	
III–IV	1.252 (0.418–3.75)	0.688	0.736 (0.076–7.147)	0.791	0.451 (0.1–2.041)	0.301	–	.988
Unknown	0.707 (0.327–1.53)	0.379	0.263 (0.052–1.336)	0.107	0.815 (0.412–1.615)	0.558	0.805 (0.162–3.988)	.79
AJCC stage								
I–II	Reference		Reference		Reference		Reference	
III–IV	0.89 (0.26–3.038)	0.852	–	0.988	0.318 (0.042–2.382)	0.265	–	.989
Unknown	0.984 (0.505–1.917)	0.961	1.237 (0.273–5.601)	0.783	1.095 (0.604–1.982)	0.766	0.802 (0.2–3.209)	.755
T stage								
T1–2	Reference		Reference		Reference		Reference	
T3–4	1.443 (0.528–3.944)	0.475	1.591 (0.165–15.308)	0.688	2.888 (0.848–9.838)	0.09	–	.989
Unknown	0.966 (0.487–1.917)	0.921	0.912 (0.183–4.55)	0.911	1.356 (0.737–2.496)	0.327	0.992 (0.247–3.974)	.991
N stage								
N0	Reference		Reference		Reference		Reference	
N1–2	0.859 (0.117–6.312)	0.881	–	0.99	–	0.976	–	.991
Unknown	0.928 (0.386–2.23)	0.867	0.785 (0.094–6.572)	0.824	0.721 (0.305–1.705)	0.457	0.711 (0.087–5.803)	.75
M stage								
M0	Reference		Reference		Reference		Reference	
M1	–	0.976	–	0.991	0.742 (0.102–5.417)	0.769	–	.767
Unknown	0.997 (0.389–2.559)	0.996	2.542 (0.491–13.172)	0.266	0.383 (0.092–1.588)	0.186	–	.528
Surgery								
Performed	Reference		Reference		Reference		Reference	
Unperformed	0.528 (0.127–2.198)	0.38	–	0.57	0.939 (0.336–2.625)	0.904	–	.553

HR: hazard ratio; CI: confidence interval; AJCC: American Joint Committee on Cancer; T: Tumour; N: Node; M: Metastasis.

## Discussion

In this study, we included 289 hidradenocarcinoma patients from the SEER database diagnosed between 2000 and 2018. This is currently the largest study on the clinicopathological features and prognosis of hidradenocarcinoma. Considering the differences in the extent of surgical resection of hidradenocarcinomas in the head and neck and other sites, we divided the population into two groups: head and neck hidradenocarcinoma and trunk and limbs hidradenocarcinoma for comparison. The results showed that there were no significant differences between the two groups in clinicopathological features and prognosis. However, the incidence of hidradenocarcinoma is higher in the elderly. In addition, if unknown patients were excluded, it could be seen that most hidradenocarcinomas had good histologic grades (I–II) and AJCC stages (I–II) at the time of diagnosis.

Previous studies have concluded that hidradenocarcinoma has high rates of recurrence and metastasis, resulting in an unfavourable prognosis [15,19]. However, our research results show that the 10-year OS rate and CSS rate of hidradenocarcinoma reach 60.2% and 90.5%, respectively. Compared with most other malignant tumours, hidradenocarcinoma has a great advantage in prognosis. The OS rate of hidradenocarcinoma is worse than the CSS rate due to the high proportion of elderly patients leading to more noncancer deaths. The recurrence-free survival rate cannot be assessed due to the lack of relevant data in the SEER database. In addition, 248 included patients had a clear AJCC M stage, of which only six patients had distant metastases, accounting for 2.4%. Hidradenocarcinoma does not have a high rate of metastasis, as reported by Souvatzidis et al. [15]. The results from survival analysis showed that age is a factor related to patient OS. Elderly patients had worse OS rates, but there was no difference in CSS rates. This is also due to the high proportion of elderly patients leading to more noncancer deaths. In addition, tumour size is a significant prognostic factor related to both OS and CSS and is inversely proportional to survival time. This is consistent with the findings of Souvatzidis et al. [15].

Although there are generally accepted histological criteria for the diagnosis of hidradenocarcinoma [8–10], some lesions do not meet all criteria. In addition, atypical hidradenomas have some focal atypical features, including lack of circumscription, nuclear pleomorphism, focal necrosis, and prominent mitotic activity [10]. Hyaloid stroma, duct formation, clear cell change, epidermoid morphology, nuclear pseudo inclusion, and nuclear groove can be observed in most atypical hidradenomas and hidradenocarcinomas [16]. Therefore, the differential diagnosis of hidradenocarcinoma is challenging. Nazarian et al. [16] compared the histological appearance of 15 atypical hidradenomas and 15 hidradenocarcinomas and proposed some distinguishing histological features of hidradenocarcinoma, including invasive growth pattern, deep extension, necrosis, nuclear pleomorphism, and ≥ 4 mitoses per 10 high-power fields. Furthermore, they detected the molecular biological alterations and found that lesions with Ki-67 > 11% and/or PHH3 > 0.7% would likely be malignant, and Her2/neu was rarely overexpressed in hidradenocarcinomas. Kazakov et al. [4] demonstrated that hidradenocarcinomas exhibit some microscopic heterogeneity, which can be classified into low-grade and high-grade. High-grade hidradenocarcinomas are histologically similar to hidradenomas. They also detected the molecular biological alterations of hidradenocarcinomas and found that both t (11;19) translocation and Her2/neu gene amplification were rare, the rate of p53 protein expression at the immunohistochemical level was high, and the frequency of TP53 mutations was relatively low [4]. Obaidat et al. [20] also reported some histological features of hidradenocarcinomas: hidradenocarcinoma cells exhibit obvious pleomorphism and an expansive lobular growth pattern accompanied by tubular differentiation, forming a large number of duct structures; the nucleus is monomorphic with high pigment and active mitosis; the cytoplasm is almost invisible, and tubular vacuoles can be seen in the cytoplasm when the tumour is necrotic.

Limited information about the clinical treatment and outcome of hidradenocarcinoma was mentioned in the literature, which contains few cases [11,15,21]. At present, early extensive surgical resection is still the first choice of treatment, but hidradenocarcinoma has an extremely obvious trend of recurrence and metastasis after extensive resection [21]. A study decades ago reported that after surgical resection, 50% of hidradenocarcinomas had local recurrence, and 60% of hidradenocarcinomas had distant metastasis [11]. In the study of Souvatzidis et al. [15], six of the seven hidradenocarcinoma cases relapsed after resection, and two cases relapsed again after the second resection. The effect of adjuvant treatment of hidradenocarcinoma needs to be verified by large-scale clinical trials [22]. Radiotherapy is generally considered beneficial for improving the local control rate [15,23]. Chemotherapy seems to be unsuitable as an adjuvant treatment for hidradenocarcinoma because no benefit was seen among patients who received adjuvant chemotherapy in the published literature [15,24–26].

Two shortcomings of the present study should be pointed out. First, there is a lack of histologic review and confirmation of the diagnosis. Because hidradenocarcinoma is a rare malignancy, histological review and confirmation of the diagnosis can help to improve the diagnostic criteria and explore more prognostic factors. Second, there is a lack of information on tumour size, grade, AJCC stage, T stage in around 50% of patients. This shortcoming leads to a reduction in the actual sample size in the survival analysis of these clinicopathological variables.

## Conclusion

Our research shows that hidradenocarcinoma is a malignant tumour with a good prognosis, which is different from previous views. Tumour size is inversely proportional to patients’ overall survival time, affecting the OS and CSS of patients. At the time of diagnosis, the tumour size of most patients is within 20 mm. This may also be one of the reasons for the good prognosis. Hidradenocarcinoma originates from the skin and is usually found at an early stage. However, because of the slow progress, some patients ignored it and missed the opportunity for early treatment. Therefore, improving health awareness, initial histological examination, and timely surgery are the keys to improving prognosis.

## Data Availability

The datasets generated during the present study are available in the official software. SEER* Stat version 8.3.9.1 repository (https://seer.cancer.gov/data/).

## Abbreviations

SEER: Surveillance, Epidemiological and End Results
AJCC: American Joint Committee on Cancer
OS: overall survival
CSS: cancer-specific survival
HRs: hazard ratios
CIs: confidence intervals.
